# Environmental Impact Assessment and End-of-Life Treatment Policy Analysis for Li-Ion Batteries and Ni-MH Batteries

**DOI:** 10.3390/ijerph110303185

**Published:** 2014-03-18

**Authors:** Yajuan Yu, Bo Chen, Kai Huang, Xiang Wang, Dong Wang

**Affiliations:** 1 Beijing Key Laboratory of Environmental Science and Engineering, School of Chemical Engineering and Environment, Beijing Institute of Technology, Beijing 100081, China; E-Mails: 1990102683@bit.edu.cn (B.C.); wx@bit.edu.cn (X.W.); guanerguan@gmail.com (D.W.); 2 College of Environmental Science and Engineering, Beijing Forestry University, Beijing 100083, China

**Keywords:** Li-ion battery, Ni-MH battery, Life Cycle Assessment, Eco-indicator 99, cycle, recycle, incineration

## Abstract

Based on Life Cycle Assessment (LCA) and Eco-indicator 99 method, a LCA model was applied to conduct environmental impact and end-of-life treatment policy analysis for secondary batteries. This model evaluated the cycle, recycle and waste treatment stages of secondary batteries. Nickel-Metal Hydride (Ni-MH) batteries and Lithium ion (Li-ion) batteries were chosen as the typical secondary batteries in this study. Through this research, the following results were found: (1) A basic number of cycles should be defined. A minimum cycle number of 200 would result in an obvious decline of environmental loads for both battery types. Batteries with high energy density and long life expectancy have small environmental loads. Products and technology that help increase energy density and life expectancy should be encouraged. (2) Secondary batteries should be sorted out from municipal garbage. Meanwhile, different types of discarded batteries should be treated separately under policies and regulations. (3) The incineration rate has obvious impact on the Eco-indicator points of Nickel-Metal Hydride (Ni-MH) batteries. The influence of recycle rate on Lithium ion (Li-ion) batteries is more obvious. These findings indicate that recycling is the most promising direction for reducing secondary batteries’ environmental loads. The model proposed here can be used to evaluate environmental loads of other secondary batteries and it can be useful for proposing policies and countermeasures to reduce the environmental impact of secondary batteries.

## 1. Introduction

Secondary batteries, also known as rechargeable batteries, are a group of batteries that can be used after discharge by charging the active substances. As they can be used repeatedly, the secondary batteries’ lives are longer than those of primary batteries. As a result, the use of secondary batteries has the potential advantages of conserving resources and reducing waste. Moreover, given their high capacity and high energy density, secondary batteries are widely used throughout the World [1,2].

However, these environmentally friendly features do not imply that secondary batteries have no impact on the ecological environment. Different types of secondary batteries have different potential effects on resources, ecosystems and human health during their production, use, disposal, or recycle stages. For example, the production and disposal phases of heavy metals, which secondary batteries contain, can be hazardous to the environment [3,4,5] due to the high worldwide levels of battery consumption. Meanwhile, material production has increased resource depletion. These metal elements and other poisonous substances could become harmful to the ecosystem if a battery is arbitrarily discarded or improperly buried.

To date, the environmental effects of secondary batteries, their resource pressures, their potential risks and hazards have not attracted enough or sufficient attention. More attention should be paid to the environmental characteristics of secondary batteries as the ecological impacts of electronic products come into focus [6]. Environmentally friendly secondary batteries will lead the development of the battery industry; therefore, an appropriate method to analyse the environmental impact of secondary batteries is necessary.

Various tools have been developed to evaluate the environmental impacts of different systems [7,8,9,10,11,12]. Life Cycle Assessment (LCA) is a suitable tool for calculating the environmental impact of certain electric products [13,14,15]. As with any product, the environmental impact of an electric product over its entire life cycle is affected by many factors. For secondary batteries, LCA analysis should consider pollution emissions and the environmental impact of producing, recycling and disposing of the batteries in a life cycle.

To reveal the environmental impact of secondary batteries, Matheys compared the environmental indicators of five electric vehicle batteries using the LCA method [16]. Zackrisson *et al.* performed a LCA study of two lithium-ion batteries to optimize the design of lithium-ion batteries for plug-in hybrid electric vehicles [17]. The LCA results of three batteries (nickel metal hydride, nickel cobalt manganese lithium-ion, and iron phosphate lithium-ion batteries) for plug-in hybrid and full performance battery electric vehicles were presented in Guillaume Majeau-Bettez’s study [3]. Longo *et al.* assessed the energy and environmental impacts of sodium/nickel chloride batteries [18]. In resource recovery, Carl found that the power and energy consumption in the production stage decreased from 43% to 8% while the proportion of recycled lead increased from 50% to 99%. That study also noted that resource consumption, such as land use by a single cycle, may be reduced by an increase in the number of cycles [19]. Slack [20] focused on the source, flow and disposal methods of the UK’s used household batteries, while Panero [21] studied the damage that these batteries caused to human health and the ecosystem after they were buried in a landfill.

The existing research has built a good foundation for the further study of the secondary batteries’ environmental impact. The extant research considers limited aspects of the relation between environmental impact and the end-of-life treatment. Dewulfa *et al.* performed a critical analysis of natural resource savings in lithium ion battery recycling [22]. However it didn’t have enough details of disposal in the end-of-life treatment and how disposal acts on the environmental impact. Based on existing research, we conducted a comprehensive assessment model to include recycle and waste stages of secondary batteries in the whole life cycle. The charge-discharge cycles were also introduced in this study to confirm how the cycles influence the LCA results and the end-of-life treatment. Through the integrated use of the Eco-indicator 99 method and several developed tools such as MATLAB and some LCA software, a quantitative model for secondary batteries’ environmental impact and end-of-life treatment analysis was applied, which was used on two types of secondary batteries. This model analyses the environmental impact of secondary batteries, and it offers suggestions to reduce the environmental impact of secondary batteries. 

The increasing public concern about the environment has resulted in stricter regulations worldwide on spent portable batteries related to the adequate destination of hazardous residues [4]. The environmental impact of secondary batteries shown from the LCA cases can help verify the chances and possibility that the policies prompt society to improve end-of-life treatment in order to treat these types of residues since the consumption of batteries is considerable around the World. 

## 2. Materials and Methods

The analysis was carried out following the ISO 14040 [23] and ISO 14044 [24] series, which are the basic standards providing a procedure to carry out a LCA. In our framework, the battery system and environmental impact assessment will be included in a LCA following the ISO 14040/14044 guidelines. 

### 2.1. LCA Model

The goal and scope and inventory of a LCA study depend on the intended use of the study. The LCA model was built on a Li-ion battery and a Ni-MH battery based on a theoretical design. Material needs were based on laboratory tests and literature references. The main goal of this LCA study was the assessment of the environmental impact of different secondary batteries made from laboratories, as well as the comparison and analysis of the impact factors from different batteries. Furthermore, it highlights the environmental points within end-of-life treatment analysis.

*Functional Unit*. The assessment objects of this study were secondary batteries. Two types of experimental batteries were selected: (1) A type of Li-ion battery (cathode material: LiNi_l/3_Co_l/3_Mn_l/3_O_2_ + 1% Fe_3_O_4_, which was considered to be the most promising material to replace LiCoO_2_ as a cathode material for Li-ion batteries) and (2) a type of Ni-MH battery (anode material: LaMg_12_ + 200% Ni alloy, which had a high first discharge capacity). During the batteries’ production processes, the types and quantities of their raw materials were analysed and recorded. The functional unit was defined as a battery whose specific capacity is 1,000 mAh/g under sustained charge cycles. All figures for environmental impact were related to one battery under the functional unit conditions.

**Figure 1:**
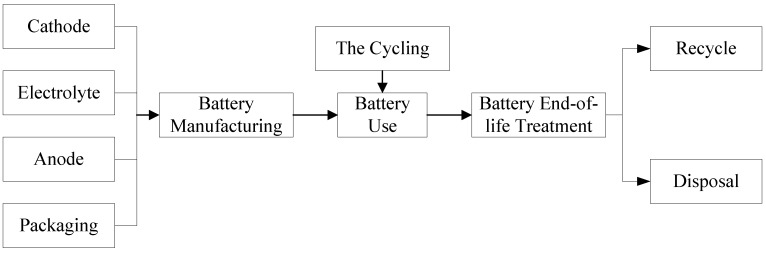
Flow diagram of the battery system.

*System boundaries*. The system boundaries were based on the general rules of ISO 14040 and 14044. This study focused on secondary batteries. The product system includes production, use and recycling. Metals and other materials will be recycled as raw materials. The battery charger and other hardware were outside the system boundary. Only the battery itself and its casing were considered. Figure 1 presents the system definition. Because there were different ways of using batteries, short transports were assumed in the use phase. Long transports were assumed in battery manufacture and the end-of-life treatment. 

*Life cycle inventory*. The life cycle inventory of the components and materials included the LiNi_l/3_Co_l/3_Mn_l/3_O_2_ + 1% Fe_3_O_4_ ternary cathode material Li-ion battery and the LaMg_12_+200% Ni alloy anode material Ni-MH battery. The first discharge-specific capacities of the LiNi_l/3_Co_l/3_Mn_l/3_O_2_ + 1% Fe_3_O_4_ ternary cathode material Li-ion battery and the LaMg_12_ + 200% Ni alloy anode material Ni-MH battery, both produced in the laboratory, were 170.9 mAh/g and 932.8 mAh/g, respectively. The types and mass of the main raw materials and energy consumption are shown in Table 1 and Table 2. The masses of raw materials and the charge-discharge data were primary data and some energy data in the battery manufacturing was secondary data. 

*Environmental impact assessment.* In line with the recommendations in Eco-indicator 99 system [25], three mainly environmental damage categories were calculated: Human health damage; Ecosystem quality impact; Resource consumption. 

**Table 1:** The inventory of main raw materials (kg) and energy consumption for the Li-ion battery (LiNi_l/3_Co_l/3_Mn_l/3_O_2_ + 1% Fe_3_O_4_, 170.9 mAh/g).

Raw material	Mass/g	w%	Energy	
Li	1.75E−05	69.63%	Fossil fuels (Heat coal in industrial furnace)	0.190 MJ
Ni	1.25E−06	4.95%	Electricity from grid	3.77 E−04 kWh
Mn	1.50E−06	5.97%		
Co	1.60E−06	6.37%		
Fe_3_O_4_	7.40E−08	0.29%		
Acetylene black	1.01E−06	4.02%		
PVDF	5.05E−07	2.01%		
LiPF_6_/PC-DMC (1 mol·L^−1^)	1.70E−06	6.76%		
Total	2.51E-05	100%		

**Table 2:** The inventory of main raw materials (kg) and energy consumption for the Ni-MH battery (LaMg_12_ + 200% Ni alloy, 932.8 mAh/g).

Raw material	Mass/g	w%	Energy	
La	3.21E−05	0.09%	Fossil fuels (Heat coal in industrial furnace)	24.6 MJ
Mg	6.75E−05	0.19%	Electricity from grid	2.95 kWh
Ni	3.70E−04	1.03%		
C	3.10E−04	0.86%		
KOH	3.36E−02	93.65%		
LiOH	1.50E−03	4.18%		
Total	3.59E−02	100%		

### 2.2. Choices and Assumption of the Study

The Eco-indicator methodology that is used to calculate the standard values conforms well to the ISO 14042 standard on Life Cycle Impact Assessment (LCIA), although some details will perhaps deviate. The LCA method was selected to comprehensively evaluate the environmental impact of secondary batteries. 

In the Life Cycle Impact Assessment process of LCA, the Eco-indicator 99 system was chosen. The Eco-indicator 99 LCIA method is based on the principles of environmental damage. The term “damage” includes 11 aspects, which can be grouped according to three main types of damage (Table 3). Standard Eco-indicators are numbers that express the total environmental load of a product or process. The core of this method is the weighing system between the different environmental aspects. The Eco-indicator point (Pt) is representative for one thousandth of the yearly environmental load of one average European inhabitant. The indicators can be calculated with appropriate LCA software. SimaPro 7.1.8 [26] was chosen as the LCA software in this study. The Eco-indicator of a material or process is a non-unit number indicating the environmental impact of the material or process based on data from a LCA tool, where a higher indicator means a greater environmental impact [27,28].

**Table 3:** The 11 “damages” grouped in three main types.

Main Types of Damage	Eco-indicator Damage
(A) human health damage	(1) carcinogens, (2) respiratory organics, (3) respiratory inorganics, (4) climate change, (5) radiation, (6) ozone layer;
(B) ecosystem quality impact	(7) eco-toxicity, (8) acid rain / eutrophication, (9) land use;
(C) resource consumption	(10) minerals, (11) fossil fuels.

### 2.3. Data Quality and Assumptions

Material synthesis and the assembly of batteries were implemented in the Beijing Institute of Technology under quality assurance conditions. The raw material masses and the charge-discharge data were measured in the lab. Energy consumption and environmental emission were collected from databases and data from the scientific literature [29]. The resource and energy flow data of the system were obtained from the data of experimental batteries and the industrial process information about raw material production. Data for the disposal and other end-of-life treatment originated from the Ecoinvent database v2.2 [30] as a background system. The environmental impacts of PVDF and LiPF_6_/PC-DMC were ignored after considering the lack of data in the Ecoinvent database for the relevant components. Acetylene black was mainly composed of carbon, and its environmental impact is mainly caused by carbon. The environmental impact of carbonyl nickel was represented by nickel sediment.

### 2.4. Quality Standardisation of Raw Materials

For the Eco-indicator comparison of the two selected batteries, it was necessary to determine a unified functional unit and standardized inventory data. Quality standardisation of the raw materials was then performed in accordance with the standard. 

In this paper, the functional unit is a battery whose specific capacity is 1,000 mAh/g. The cycles are the times a consumer charges and discharges a battery in the use phase. The requirements of the use step impact the charge-discharge cycles. Each battery requires a material independently from the use step. The needs are related to the choice of the functional unit for two different batteries. Depending on the relationship between the charge-discharge cycles and the specific capacity, the material needs can be related with the use step. And this would be reflected in the life cycle inventory. 

Based on the condition of battery tests in the laboratory, the total capacity of batteries can be calculated by Equation (1) in which the cycle was *N_k_*:

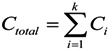
(1)
where *C_i_* can be calculated by fitting the formula obtained through curve fitting of the cycle performance.

To facilitate the comparison of results, the mass of raw material needs (*m_s_*) under standard specific capacity (*C_s_* = 1,000 mAh/g) can then be calculated by Equation (2):

*m_s_* = *C_s_* · *m*/*C_total_* (2)

where *m* is the battery mass under test, and *m_s_* is the result of quality standardisation of raw materials for the functional unit. Through this calculation method, a life cycle inventory of the raw materials was assembled. 

### 2.5. End-of-Life Treatment Policy Analysis

End-of-life treatment, such as disposal and recycle, contributes to the environmental impact of batteries [31]. Different measures for treatment and the proportion of different measures lead to changes in different environmental impact categories. With the Eco-indicator 99, the total environmental impact of a unit mass of raw materials during stages of production, use, disposal and recycle was quantified. Incineration (while mixed with other types of wastes) and landfill are the common means of disposal for batteries. Due to China’s waste treatment trends, we selected incineration as the typical means of disposal for discarded batteries. Depending on the Eco-indicator points and their changing with different end-of-life treatment, it can be shown how recycle and disposal affect the environment and what level the environmental impact has.

By means of the slice function in MATLAB [32], the Eco-indicator distribution obtained above is shown in the cycles, incineration rate and recycle rate in three-dimensional space and two-dimensional space composed of any two of the above three factors. End-of-life analysis was performed based on these slice graphs. The analysis can be used to support policies concerning the end-of-life treatment.

### 2.6. Evaluation Procedures

**Figure 2:**
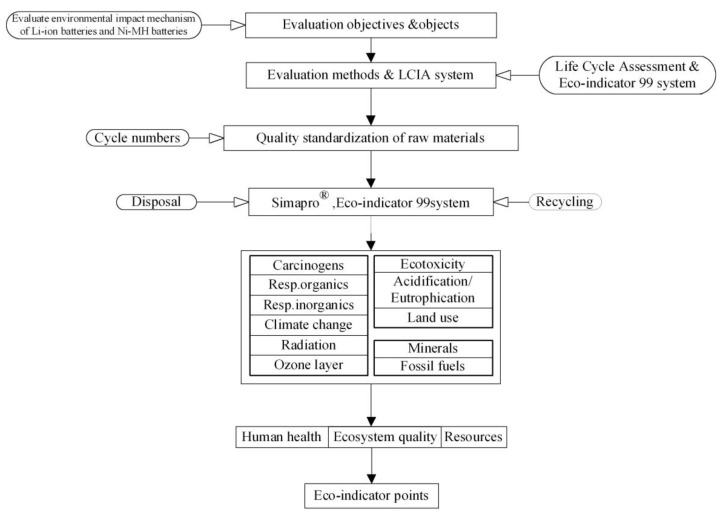
Research steps of the secondary battery environmental impact assessment.

Figure 2 shows the research scheme of our environmental impact and end-of-life treatment policy analysis of secondary batteries. 

The type and quantity of raw material data were analysed using the SimaPro7.1.8. Finally, with the Eco-indicators of each ingredient, the Eco-indicator distributions of secondary batteries with influencing factors (cycle number, incineration rate and recycle rate) were obtained. For discarded batteries, incineration and recycle were chosen to be the end-of-life treatment measures. 

## 3. Results and Discussion

### 3.1. Cycle Performance Fitting

**Figure 3:**
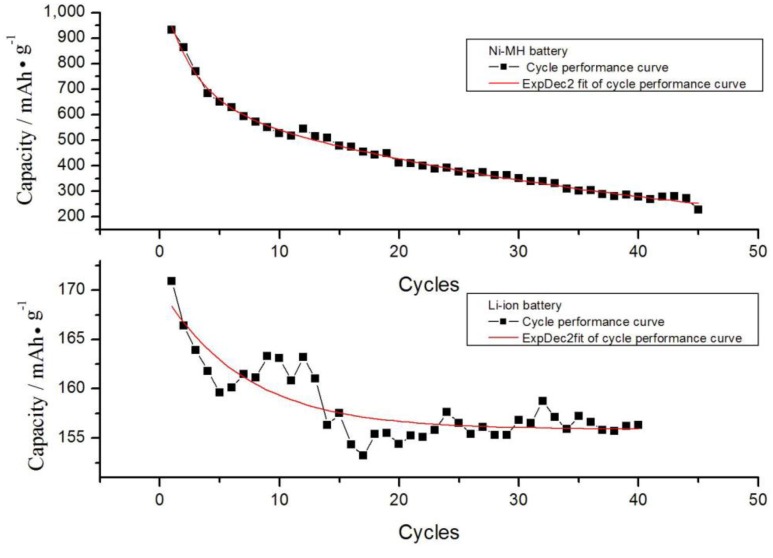
Cycle performance fits of the selected Ni-MH battery & Li-ion batteries.

The cycle performance of the selected batteries is shown in Figure 3. Exponential decay fitting was performed. The fitting equation of the red curves is as follows:

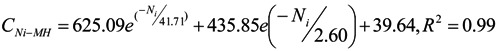
(3)

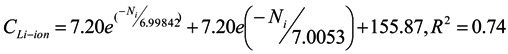
(4)


The results were obtained in accordance with the method described in Section 2.1 and Section 2.4.

### 3.2. Comparison of Selected Batteries

#### 3.2.1. Three-Dimensional Eco-Indicator Slices of the Two Selected Batteries

**Figure 4:**
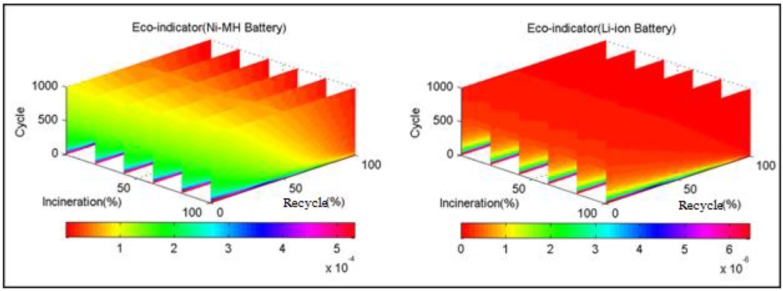
Three-dimensional slices of eco-indicator of the two selected batteries.

Three-dimensional Eco-indicator slices of the two selected batteries are shown in Figure 4. Here, the three axes represent the cycle number, incineration rate and recycle rate of the corresponding batteries. The Eco-indicator point of batteries is indicated by different gradient colours. The Eco-indicator point of the selected Li-ion battery is significantly lower than that of the selected Ni-MH battery. For further analysis of the influence of the three factors on the Eco-indicator of the two selected batteries, the Eco-indicator distribution in two-dimensional space composed by any two of the three factors were drawn based on Figure 4.

#### 3.2.2. Influence of Cycles and Incineration Rate on Eco-Indicator Distribution

**Figure 5:**
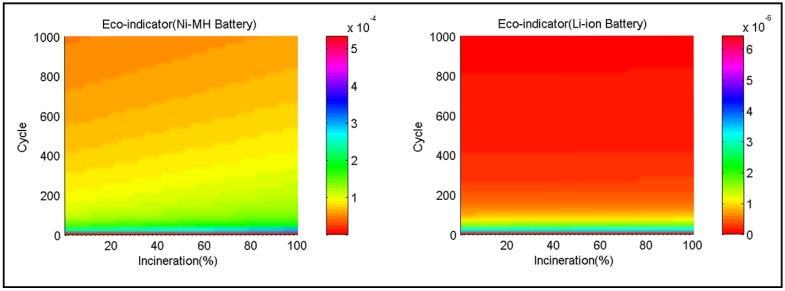
Influence of cycles and incineration rate on the Eco-indicator of the two selected batteries.

The influence of cycles and incineration rate on the Eco-indicator distribution of two selected batteries at a certain recycle rate (50%) is shown in Figure 5. As seen, the Eco-indicator points of the two selected batteries decrease rapidly with the increase in cycles when the number of cycles is less than 200, but its attenuation is less obvious after 200 cycles. Incineration rate has a greater impact on the Eco-indicator point of the selected Ni-MH battery, and the impact increases after the incineration rate reaches 50% or more. In Li-ion batteries, the Eco-indicator point is scarcely affected by the incineration rate, as shown in the figure. 

#### 3.2.3. Influence of Cycles and Recycle Rate on Eco-Indicator Distribution

**Figure 6:**
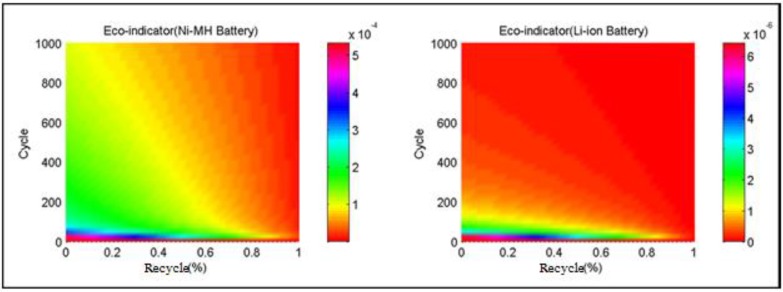
Influence of cycles and recycle rate on the Eco-indicator of the two selected batteries.

Figure 6 shows the influence of cycles and recycle rate on the Eco-indicator distributions of the two selected batteries when the incineration rate is 50%. The Eco-indicator points decrease rapidly when the cycle numbers are less than 200. The points of the two selected batteries decay with the increase of recycle rate, particularly when the rate is above 50%. The Eco-indicator point of Ni-MH battery has a larger attenuation. The influence of recycle rate is more obvious, while the influence on the Li-ion battery can be clearly observed when the recycle rate reaches 40%–50%.

#### 3.2.4. Influence of Recycle Rate and Incineration Rate on Eco-Indicator Distribution

**Figure 7:**
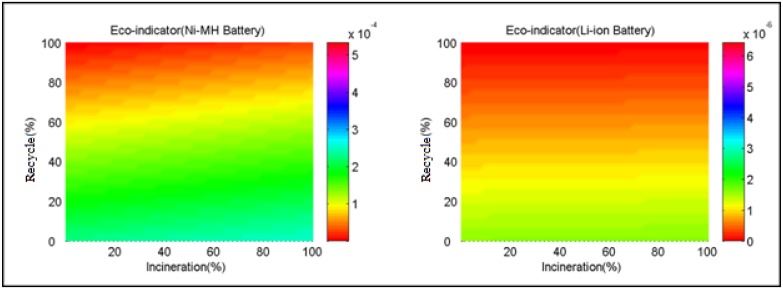
Influence of recycle rate and incineration rate on the Eco-indicator of the two selected batteries.

Figure 7 shows the effect of recycle rate and incineration rate on the Eco-indicator of the two selected batteries when the cycle number is 100. As confirmed by this diagram, the Eco-indicator points of the two selected batteries decay with the increase of recycle rate and incineration rate. These two factors have a greater impact on the Eco-indicator of the Ni-MH battery than the Li-ion battery; in contrast, their impact on the Li-ion battery is small, and the Eco-indicator point of the Li-ion battery is nearly unaffected by the incineration rate.

In summary, to reduce the environmental impact of selected batteries, the charge-discharge cycle number during the use stage should be at least 200. Battery incineration has a greater impact on the Eco-indicator point of the Ni-MH battery, while its impact on the Li-ion battery is small. Battery recycling can reduce the battery’s environmental impact, particularly for Ni-MH batteries, and the incineration has little effect on reducing the environmental impact of Ni-MH batteries.

### 3.3. Uncertainty Analysis

Based on the laboratory battery test conditions, the charge-discharge cycle numbers of the batteries and the masses of raw materials were the input data of the LCA. According to the quality standardisation of the raw materials’ masses, the cycle number of the batteries was assumed to be normally distributed under 95% confidence interval. The distributions presented the impact of uncertainty data. For the Ni-MH battery, the Eco-indicator point ranges from 0.5 × 10^−4^ Pt to 2.0 × 10^−4^ Pt when the charge-discharge cycle number ranges from 0 to 1,000. For the Li-ion battery, the Eco-indicator point ranges from 0.1 × 10^−4^ Pt to 1.0 × 10^−4^ Pt when the charge-discharge cycle number ranges from 0 to 1,000. According to the results, if the battery has a short use phase, the environmental impact will increase. If secondary batteries are under unsteady use conditions, they always have a short use phase and short charge-discharge cycles. Since the cycle numbers are related with energy consumption, the uncertainty of energy consumption in battery use phase has larger impacts on the Li-ion battery. Improving the cycle performance of the Li-ion battery can efficiently reduce the environmental impact. 

## 4. Conclusions and Policy Implications

In this paper, an assessment framework was applied to conduct an environmental impact and end-of-life treatment analysis for secondary batteries based on the Eco-indicator 99 system. This applied model comprehensively evaluated the cycle, recycle and waste (for example, incineration) stages of secondary batteries. The analysis of the end-of-life treatment is helpful to improve some extant policies concerning secondary batteries. Two types of secondary batteries were studied by means of life cycle assessment. The results show that: (1) The Eco-indicator points of the two selected batteries decrease rapidly with the increase of the cycle number up to 200, but the attenuation is small after 200; (2) the incineration rate has greater impact on the Eco-indicator point of the Ni-MH battery than of the Li-ion battery, so policies and regulations concerning the disposal of the two kinds of batteries should be different; (3) the Eco-indicator points of the two types of batteries decay with the increase of the recycle rate, which means a strict recycle policy should be set; and (4) the influence of the recycle rate on the Ni-MH battery is more obvious, while the influence on the Li-ion battery can be clearly observed when the recycle rate is 40%–50%.

Due to the assessment of environmental loads about the selected secondary batteries, several policy implications should be considered for reducing environmental impact of secondary batteries. First, policy-makers should encourage the use of higher energy density batteries with better chemical characteristics. Additional cycles in a longer service life will result in lower environmental loads. To reduce the environmental impact of selected batteries, the charge-discharge cycle number during the use stage should be at least 200. In other words, according to the assessment, a policy should be proposed to require a minimum of 200 cycles for secondary batteries. 

Second, secondary batteries should be sorted out from refuse because their contents and properties are different from those of common household garbage. For instance, most batteries contain heavy metals, such as cobalt and nickel, as well as some organic pollutants. Environmental risks will increase if secondary batteries are buried or combusted with mixed municipal waste. 

Third, end-of-life treatment policies for secondary batteries should be carefully selected. For example, incineration measures should be put in place for Ni-MH batteries, and battery recycle can significantly reduce the environmental impact of Ni-MH batteries. Additionally, the recycle of Li-ion batteries may result in higher efficiency and lower environmental loads. In summary, the mechanism analysis tools proposed in this paper can also be used to evaluate the environmental impact of other secondary batteries, and the policies proposed in this paper may be useful in setting measures to reduce the environmental impact of these batteries.

## Nomenclature

*C_i_*	battery charge-discharge capacity	LCIA	life cycle inventory assessment
*C_total_*	total battery capacity in use phase	*m*	battery mass under test
*C_s_*	standard specific capacity per functional unit	*m_s_*	required raw materials mass per functional unit
LCA	life cycle assessment	*N_k_*	charge-discharge cycles in use phase
